# Systematic analyses uncover robust salivary microbial signatures and host-microbiome perturbations in oral squamous cell carcinoma

**DOI:** 10.1128/msystems.01247-24

**Published:** 2025-01-28

**Authors:** Zewen Han, Yichen Hu, Xin Lin, Hongyu Cheng, Biao Dong, Xuan Liu, Buling Wu, Zhenjiang Zech Xu

**Affiliations:** 1 Shenzhen Clinical College of Stomatology, School of Stomatology, Southern Medical University 620603 , Shenzhen, Guangdong, China; 2 Shenzhen Stomatology Hospital (Pingshan) of Southern Medical University 47861 , Shenzhen, Guangdong, China; 3 Center of Stomatology, The Second Affiliated Hospital, Jiangxi Medical College, Nanchang University, Nanchang, Jiangxi, China; 4 JXHC Key Laboratory of Periodontology, The Second Affiliated Hospital of Nanchang University, Nanchang, Jiangxi, China; 5 The Institute of Periodontal Disease, Nanchang University, Nanchang, Jiangxi, China; 6 State Key Laboratory of Food Science and Technology, Nanchang University, Nanchang, Jiangxi, China; 7 Department of Endodontics, Shenzhen Stomatology Hospital (Pingshan) of Southern Medical University, Shenzhen, Guangdong, China; 8 School of Stomatology, Southern Medical University, Guangzhou, Guangdong, China; 9 Stomatology Hospital Pingshan of Southern Medical University, Shenzhen, Guangdong, China; Argonne National Laboratory, Lemont, Illinois, USA

**Keywords:** oral squamous cell carcinoma, salivary microbiome, meta-analysis, age, gender

## Abstract

Oral squamous cell carcinoma (OSCC) is a prevalent malignancy in the oral-maxillofacial region with a poor prognosis. Oral microbiomes play a potential role in the pathogenesis of this disease. However, findings from individual studies have been inconsistent, and a comprehensive understanding of OSCC-associated microbiome dysbiosis remains elusive. Here, we conducted a large-scale meta-analysis by integrating 11 publicly available data sets comprising salivary microbiome profiles of OSCC patients and healthy controls. After correcting for batch effects, we observed significantly elevated alpha diversity and distinct beta-diversity patterns in the OSCC salivary microbiome compared to healthy controls. Leveraging random effects models, we identified robust microbial signatures associated with OSCC across data sets, including enrichment of taxa such as *Streptococcus*, *Lactobacillus*, *Prevotella*, *Bulleidia moorei*, and *Haemophilus* in OSCC samples. The machine learning models constructed from these microbial markers accurately predicted OSCC status, highlighting their potential as non-invasive diagnostic biomarkers. Intriguingly, our analyses revealed that the age- and gender-associated signatures in normal saliva microbiome were disrupted in the OSCC, suggesting perturbations in the intricate host-microbe interactions. Collectively, our findings uncovered complex alterations in the oral microbiome in OSCC, providing novel insights into disease etiology and paving the way for microbiome-based diagnostic and therapeutic strategies. Given that the salivary microbiome can reflect the overall health status of the host and that saliva sampling is a safe, non-invasive approach, it may be worthwhile to conduct broader screening of the salivary microbiome in high-risk OSCC populations as implications for early detection.

**IMPORTANCE:**

The oral cavity hosts a diverse microbial community that plays a crucial role in systemic and oral health. Accumulated research has investigated significant differences in the saliva microbiota associated with oral cancer, suggesting that microbiome dysbiosis may contribute to the pathogenesis of oral squamous cell carcinoma (OSCC). However, the specific microbial alterations linked to OSCC remain controversial. This meta-analysis reveals robust salivary microbiome alterations. Machine learning models using differential operational taxonomic units accurately predicted OSCC status, highlighting the potential of the salivary microbiome as a non-invasive diagnostic biomarker. Interestingly, age- and gender-associated signatures in the normal salivary microbiome were disrupted in OSCC, suggesting perturbations in host-microbe interactions.

## INTRODUCTION

The oral cavity harbors a complex and dynamic microbial ecosystem, known as the oral microbiota, which exhibits the second highest biomass after the gut microbiota (1). This intricate microbial community intimately interacts with the host through diverse microbe-host interactions (2) and plays a crucial role in maintaining oral health (3). Saliva serves as a reservoir for the oral microbiota, containing microorganisms derived from different habitats within the oral cavity, making it an optimal representative of the overall oral microbiota (4).

Oral squamous cell carcinoma (OSCC) is the predominant type of oral cancer, accounting for approximately 90% of all oral/maxillofacial cancers (5), and exhibits a markedly higher incidence and mortality among men and elderly individuals (6). A large portion of OSCC patients were diagnosed at an advanced stage and suffered from a poor prognosis (7–9).

Numerous studies have revealed associations between dysbiosis of the oral microbiota and OSCC. Oral microbiota may promote the tumorigenesis of OSCC (10, 11). Given the ease and non-invasive nature of saliva sampling, the oral microbiome holds promise as a potential tool for OSCC screening and early detection. With the advancement in sequencing technologies, multiple studies have compared the salivary microbiota between OSCC patients and healthy controls. Despite variations among studies, such as study populations, sequencing methodologies, analytical tools, and statistical approaches, they reported distinct microbiome compositions between these two groups (12–20). However, discrepancies existed regarding the detailed differences in the salivary microbiome.

Some studies indicated that the alpha diversity of oral microbes was higher in OSCC patients compared to healthy individuals (21, 22), while other studies observed a reverse trend (15, 23). Furthermore, reports on specific salivary microbial taxa associated with OSCC varied among different studies. For instance, Heng et al. (23) reported that *Neisseria*, *Aggregatibacter*, and *Capnocytophaga* genera were increased, while *Streptococcus* and *Veillonella* genera were decreased in the saliva of OSCC patients compared to the healthy controls. Zhou et al. (22) found enriched *Veillonella* and *Fusobacterium* and reduced *Streptococcus*, *Neisseria*, and *Prevotella* in OSCC salivary microbiome. Another study identified a higher relative abundance of *Streptococcus*, *Gemella*, *Granulicatella*, and *Lachnospira* in OSCC individuals (24). Recent research demonstrated increased *Streptococcus anginosus*, *Abiotrophia defectiva*, and *Fusobacterium nucleatum* and reduced *Prevotella histicola*, *Haemophilus parainfluenzae*, and *Fusobacterium periodonticum* in OSCC patients compared to healthy subjects (25). Thus, there is currently an absence of consensus regarding universal microbiome changes and microbial signatures across studies in the context of oral microbiota and OSCC.

Besides disease stages, demographic factors, such as age and gender, are also known to significantly shape the oral microbiome. During infancy and early childhood, the oral microbiome undergoes initial colonization and progressive diversification (26, 27). Puberty, pregnancy, and aging can induce further shifts in microbial composition and function due to hormonal fluctuations, altered physiology, and medication use (28–30). Computational approaches have successfully utilized oral bacterial signatures to predict an individual’s age, outperforming gut microbiome-based predictions (31). Furthermore, distinct gender-specific differences in the relative abundances of certain oral bacterial taxa have been well documented (32). Females exhibit a higher abundance of bacteria such as *Streptococcus*, *Prevotella*, and *Granulicatella* in their saliva, while males demonstrate higher proportions of other oral bacteria, including *Campylobacter*, *Veillonella*, *Porphyromonas*, and *Oribacterium* (32). This divergence in oral microbiota between genders arises in part from differences in sex hormones, which modulate immunity and resource allocation in the body (33). However, the interplay between OSCC and these demographic factors remains elusive and awaits further investigation.

In this study, we aimed to comprehensively investigate the salivary microbiome alterations associated with OSCC by pooling and meta-analyzing 16S rRNA gene amplicon sequencing data from 11 published studies (12–17, 19, 20, 22, 34, 35). Standardized microbiome pipelines and consistent statistical methods were applied to remove heterogeneity introduced by varying analytical approaches. After adjusting the batch effects, we identified consistent alterations in the saliva microbiome and reproducible salivary microbial biomarkers that could distinguish healthy and OSCC samples via machine learning classification models. Additionally, we explored the relationship between demographic factors and salivary microbiome in OSCC patients. Our findings indicate that the predictive salivary microbial signatures for age and gender, which are typically observed in healthy subjects, were disrupted within the OSCC cohorts.

## MATERIALS AND METHODS

### Study collection and sample filtering

A systematic literature search was performed in the Web of Science (WOS) and PubMed databases using the following search terms restricted in the title and abstract: (oral OR “head and neck”) AND (cancer or carcinoma or carcinogenesis or neoplasm or tumor) AND (microb* or microorgan* or bacter*) AND (saliv* or “mouth wash*” or “oral rins*” or gargl*) AND (“16S rRNA” or “16S rDNA” or metagenom* or sequenc*). This query targeted studies published between 1 January 2010 and 28 February 2023. Head and neck squamous cell carcinoma (HNSCC) were initially searched to include more OSCC samples because OSCC is the most common subtype of HNSCC. The initial search yielded 592 and 162 articles from WOS and PubMed, which were next independently screened by two researchers to ensure the inclusion of (i) 16S rRNA sequencing data and (ii) human samples from healthy/oral potentially malignant disorders (OPMD)/OSCC individuals. Non-OSCC HNSCC samples were excluded next. Raw sequencing data and metadata were downloaded from public databases or requested from authors via email, resulting in a total of 11 studies included in the meta-analysis.

### Standardized pipelines

Raw data were processed using Quantitative Insights Into Microbial Ecology 2 (QIIME2, version 2022.2) (36). Primers and Adaptors were trimmed via the “qiime cutadapt” plugin. Sequences were filtered based on the quality score using “qiime quality-filter q-score” with default parameters. Singletons, sequencing artifacts, and chimeras were next removed via the Deblur pipeline and generated an amplicon sequence variant (ASV) table with single-nucleotide resolution (37). Closed-reference operational taxonomic unit (OTU) picking method was employed to merge ASV sequences targeting different hypervariable regions from different studies. ASVs were clustered into OTUs at 99% identity against Greengenes database (version 13_8) (38) using “qiime vsearch cluster-features-closed-reference.” Taxonomy and tree files were downloaded from the Greengenes database. Samples with less than 1,000 reads were removed from the downstream analysis, resulting in 885 samples and 4,192 OTUs.

### Batch effects removal

To mitigate batch effects between different studies, the conditional quantile regression (ConQuR) approach was utilized (39). To obtain the optimal performance, we tuned the parameters using the Tune_ConQuR function with disease state as the key variable and sample type and region as the covariates. Additional biological variables were not included due to lack of availability. ConQuR-adjusted OTU table was used in the downstream analyses.

### Diversity analysis

Samples were subsampled to 27,003 reads per sample according to the rarefaction curve (Fig. S1). Alpha diversity and beta diversity were calculated via the “qiime diversity” plugin. The alpha diversity between healthy control and OSCC samples was compared as described in Bisanz et al. (40). Briefly, alpha metrics were first normalized against the geometric mean of the healthy control group within each study. *t*-tests (t.test function in R package base) were used to calculate the statistics. Random effects models (lmer function in R package lmerTest) with the formula log2(fold difference) ~ disease stage + (1|study) were applied to calculate the combined log2(fold difference). To compare the microbial composition between healthy control and OSCC samples, the adonis test (adonis function in R package vegan) was employed with 999 permutations. The combined *R*^2^ was calculated by defining the study as a strata variable to account for potential study-specific effects (40).

### Differential analysis

Differential analysis was performed as described in Shah et al. (41). Briefly, studies encompassing both healthy and OSCC samples were included in this analysis. OTUs with less than 10 reads across the data sets were removed prior to downstream assessment. The between-group comparison was accessed using the R package DESeq2 on an individual study basis (42). For OTUs present in more than half of the included data sets, we applied the rma function in the R package metafor to calculate their combined log2 (fold change) (43). *P* values were adjusted using the Benjamini–Hochberg method (p.adjust function in R package stats). OTUs with a combined false discovery rate (FDR) ≤ 0.01 were considered as differentially abundant taxa between healthy control and OSCC across studies.

### OSCC classification

All machine learning analyses were performed using the Python sklearn module (version 0.24.1) (44). Random forest (RF) algorithm, which exhibited superior performance on high-dimensional and complex microbiome data (45), was utilized to predict disease states based on microbial relative abundance profiles. To ensure the fidelity and robustness of the RF model, studies encompassing any group with a sample size below 10 were filtered from this analysis, as such limited sample sizes could potentially compromise the validity of the modeling outcomes. Ten-repeated stratified fivefold cross-validation method was used to ensure balanced healthy and OSCC samples in the training and test data. Study-to-study validation was trained on one study and tested on another. Leave-one-study-out validation first left out samples from one study as the held-out test set. The model was next trained on the remaining studies and evaluated on the hold-out study. The area under the receiver operating characteristic curve (AUROC) was used to measure the performance of the RF model.

### Age prediction

Samples were grouped by age at 5-year intervals. The healthy samples were split into training and test sets, stratified by age group. Multiple regression algorithms were benchmarked, including RF, gradient boosting, lightGBM, catBoost, XGBoost, and AdaBoost regressors. Model evaluation was based on mean absolute error (MAE) computed over fivefold cross-validation on the training set. RF regression outperformed other models. Hyperparameters were tuned using randomized search to minimize MAE. Finally, the full healthy data set was fitted to the optimized model using the repeated stratified k-fold cross-validation and validated on both the training and test healthy data sets, as well as all the OSCC samples. Since the delta age (predicted age − chronological age) was negatively correlated with chronological age (Fig. S2), a nonparametric approach was further employed to correct the regression bias as described in Ye et al. (46). Briefly, the predicted age was adjusted by subtracting the mean delta age calculated within the corresponding age group.

### Gender classification

Healthy samples were partitioned into training and test sets using 10-repeated stratified fivefold cross-validation, stratified by gender. The RF classifier was trained on the training set and tested on training, test set, and OSCC data. AUROC was used to assess the accuracy of the model.

## RESULTS

### Characteristics of studies in meta-analysis

**Fig 1:**
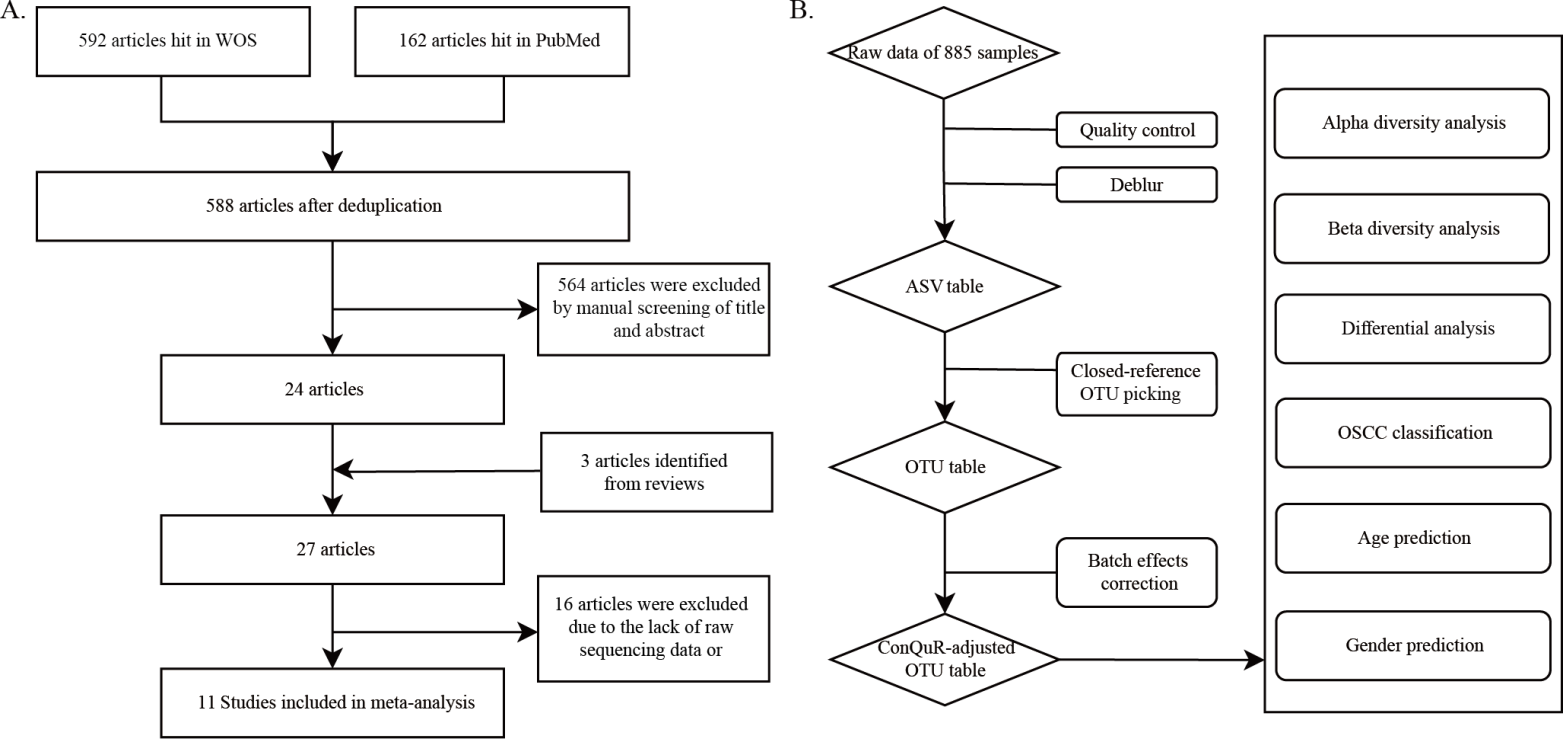
Study collection (**A**) and analysis workflow (**B**).

To investigate the alterations in the salivary microbiome during the progression of OSCC, we conducted a comprehensive literature search and manual screening (Fig. 1A; see Materials and Methods for details), resulting in the inclusion of 11 16S rRNA data sets. Our meta-analysis encompassed a total of 396 OSCC samples, 172 OPMD samples, and 317 healthy control samples (Table 1; Fig. S3A). Other sample information is provided in Table S1 and Fig. S3: there are more male samples than female samples (Fig. S3B). Most studies sequenced the V3–V4 region of the 16S rRNA gene (Fig. S3C). Samples were collected using oral rinse or saliva drooling methods (Fig. S3D). The majority of the cohorts were from the Eastern Asian population (Fig. S3E).

**TABLE 1:** Characteristics of studies included in this meta-analysis

No.	Project no.	Year of publication	Sequencing region	Sample type	Sample size			Reference
					HC	OPMD	OSCC	
1	PRJNA870048	2023	V3–V4	Oral rinse	90		91	(12)
2	PRJNA813634	2022	V4	Saliva			25	(34)
3	PRJNA756784	2022	V3	Oral rinse	39		20	(13)
4	PRJNA412445	2021	V4–V5	Saliva			17	(22)
5	PRJEB39064	2021	V3–V4	Saliva	25	21	27	(14)
6	PRJNA751046	2021	V6–V8	Oral rinse	9		9	(15)
7	PRJNA700849	2021	V4	Saliva	8		2	(16)
8	OEP000837	2021	V3–V4	Saliva	12		32	(17)
9	PRJEB37501	2021	V3–V4	Saliva		28	45	(35)
10	PRJNA421234	2018	V3–V4	Saliva	7		14	(19)
11	PRJNA386665	2017	V4	Saliva	127	123	124	(20)

### Study heterogeneity and batch effect adjustment

**Fig 2:**
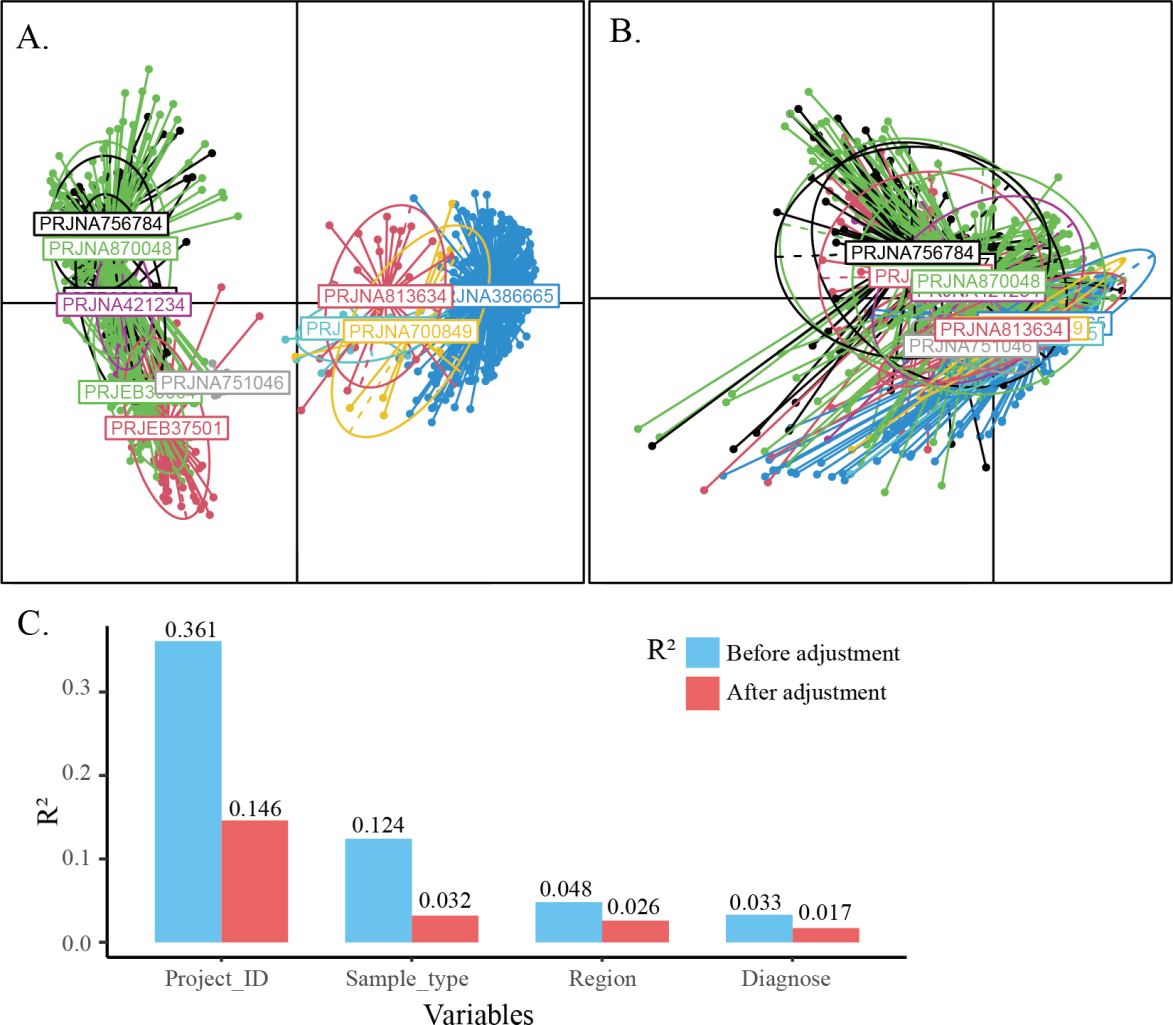
Study heterogeneity and batch effect correction. (**A**) PCoA plot showed significant differences between studies. (**B**) Different studies were aligned after ConQuR adjustment. (**C**) Adonis test evaluated the effects of various factors on the oral microbiome before and after ConQuR adjustment.

To mitigate disparities introduced by different analytical approaches, standardized bioinformatic pipelines were implemented to process the raw sequencing data across all the data sets. Nonetheless, intrinsic technical variations, such as sample collection, DNA extraction protocols, primers, and sequenced regions, still persisted across studies, which is virtually impossible to eliminate completely. The PCoA plot based on Bray-Curtis dissimilarity demonstrated significant study-specific clustering (Fig. 2A). Potential factors shaping the saliva microbiome were next assessed. The ADONIS analysis showed that the study effect was the predominant factor influencing the microbial community, accounting for 36.1% of the overall variability, followed by sample type (12.4%), geographical region (4.8%), and disease category (3.3%, Fig. 2C).

The batch effect may obfuscate genuine microbial signals between disease groups and lead to spurious conclusions, thus posing a critical challenge to downstream analyses. To address this issue, we utilized the conditional quantile regression algorithm, which is designed for microbiome data and employs a two-part quantile regression model to adjust the batch effects (39). It potentially eliminated batch-specific variations without changing the underlying biological signals. After ConQuR correction, the *R*^2^ of the study decreased from 36.1% to 14.6% (Fig. 2C), and the study-specific clusters were aligned in the PCoA plot (Fig. 2B), indicating a successful equalization of variability across batches. The variance explained by sample type and region was also reduced significantly, respectively, suggesting that ConQuR effectively minimized batch effects while preserving disease-associated microbial signatures. Therefore, the ConQuR-adjusted OTU table was used in the downstream analyses.

### Cross-study microbiome alterations and microbial biomarkers in OSCC progression

**Fig 3:**
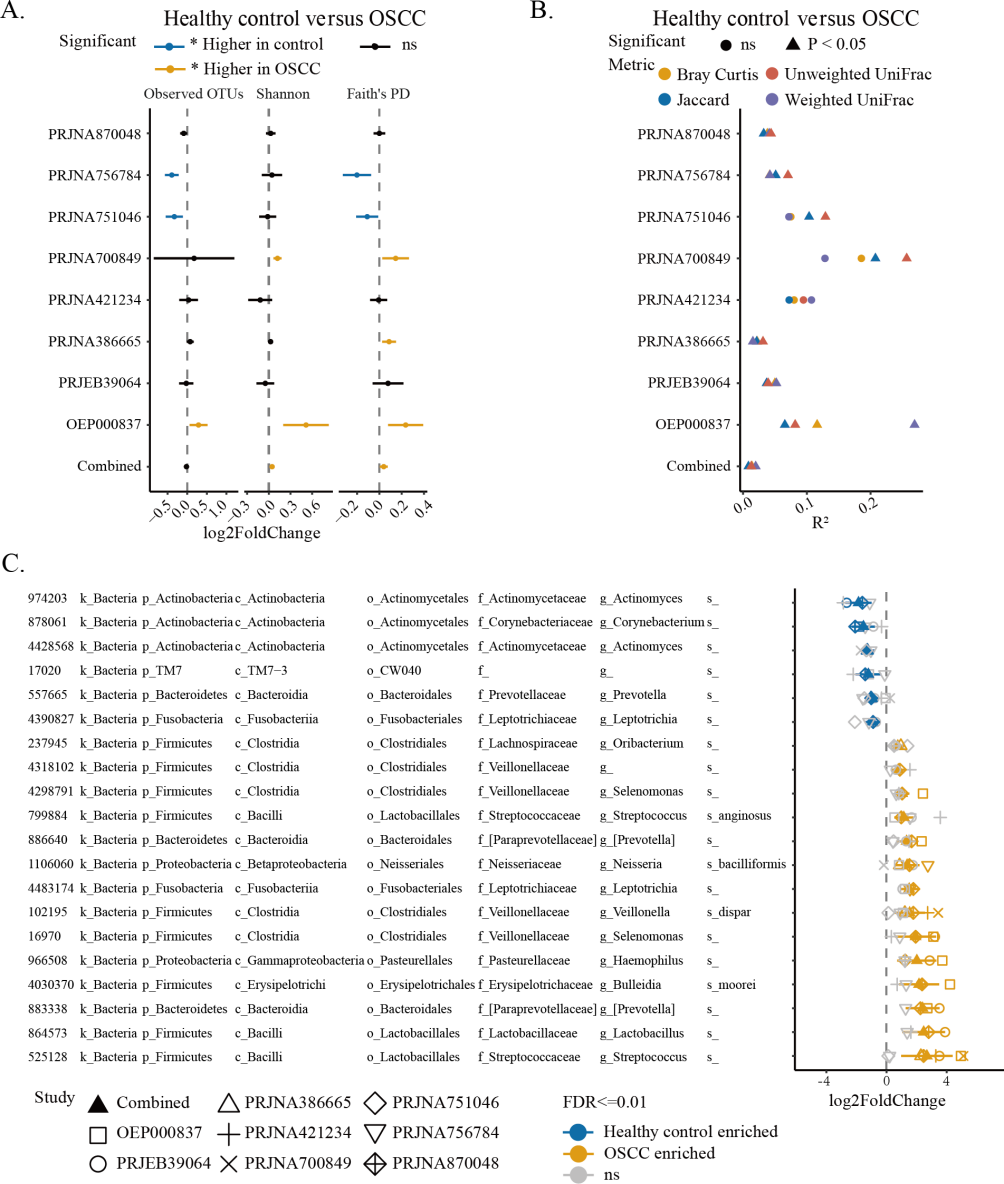
Alterations in the oral microbiome associated with OSCC across studies. (A) Forest plots depicting alpha diversity differences between healthy and OSCC samples. Dots on the left of the gray dashed line represent higher diversity in healthy groups; dots on the right indicate higher diversity in OSCC samples. Horizontal lines represent the 95% confidence intervals. (B) Differences between healthy and OSCC microbiome communities were calculated using the ADONIS analysis based on Bray-Curtis, Jaccard, unweighted UniFrac, and weighted UniFrac distance metrics. The combined *R*^2^ was computed by setting the study as the stratifying factor in the ADONIS test. (C) Top 20 differential OTUs with the largest effect sizes between healthy controls and OSCC samples.

Of the 11 studies included in the analysis, eight contained salivary samples from both control and OSCC subjects. Alpha diversity was first compared between these two groups within each individual study. A random effects model was next employed to calculate the combined statistical significance, which revealed elevated Shannon index and Faith’s PD in the OSCC patients relative to the healthy subjects, while no significant difference was identified in observed OTUs (Fig. 3A). Based on the individual data sets and combined results, it was consistently demonstrated that the overall microbiome composition differed significantly between healthy and OSCC samples (Fig. 3B). The differences between OPMD and other disease stages were also examined. Only two studies had both OPMD and healthy samples, and only three studies contained both OPMD and OSCC samples. The combined alpha diversity analysis revealed no significant differences between the OPMD and the other groups (Fig. S4A and B). In contrast, the combined beta-diversity analysis indicated significant alterations in the OPMD microbiome compared to the OSCC and healthy groups (Fig. S4C and D). Nonetheless, these results were limited by the small number of data sets, which necessitates further validation with additional data in future studies. Given the constrained data set available, we focused on the comparisons between healthy and OSCC microbial samples in subsequent analyses.

Differential abundance analysis combined with random effects models was performed to identify the reproducible microbial signatures across studies. Twenty-nine OTUs with a FDR ≤ 0.01 were considered as the significant microbial biomarkers between healthy and OSCC individuals (Fig. S5). OTUs enriched in the healthy control group were *Actinomyces*, *Corynebacterium*, CW040, *Prevotella,* and *Leptotrichia*; OTUs enriched in OSCC with the largest effect size were *Streptococcus*, *Lactobacillus*, *Prevotella*, *Bulleidia moorei,* and *Haemophilus* (Fig. 3C). We noticed that the relative abundance of 72.4% (21/29) of the differential microbes was increased at the OSCC stage compared to healthy controls (Fig. S5), indicating that the oral microenvironment of OSCC patients may have undergone alterations that facilitated the proliferation of certain microbes that typically exhibit low abundances under normal circumstances. Overall, these findings suggest that dysbiotic changes in the oral microbiome may be associated with oral carcinogenesis.

Individual studies have compared the oral microbiome obtained through various sampling methods, revealing similar microbiome profiles between saliva- and rinse-collected samples (47–50). In this meta-analysis, we also evaluated the heterogeneity between these two sample types using included data sets. We first stratified the studies by sample type and performed separate analyses. The adonis analysis showed significant differences in combined *P*-values between healthy and OSCC samples, irrespective of the sampling method used (Fig. S6A and B). To further assess the concordance in microbial changes associated with disease across sample types, we pooled all saliva microbiome samples to construct a random forest model to predict the disease status of rinse samples, achieving an AUROC of 0.83 (Fig. S6C). When using rinse microbiome data to predict disease status from saliva samples, the model yielded an AUROC of 0.70. This relatively lower predictive accuracy may be attributed to the smaller sample size of the rinse group. These findings suggest that both saliva and rinse microbiomes exhibit consistent alterations in response to OSCC. Therefore, we deem it reasonable to merge these two types of samples in the context of our meta-analysis.

### Microbial classification models for OSCC

**Fig 4:**
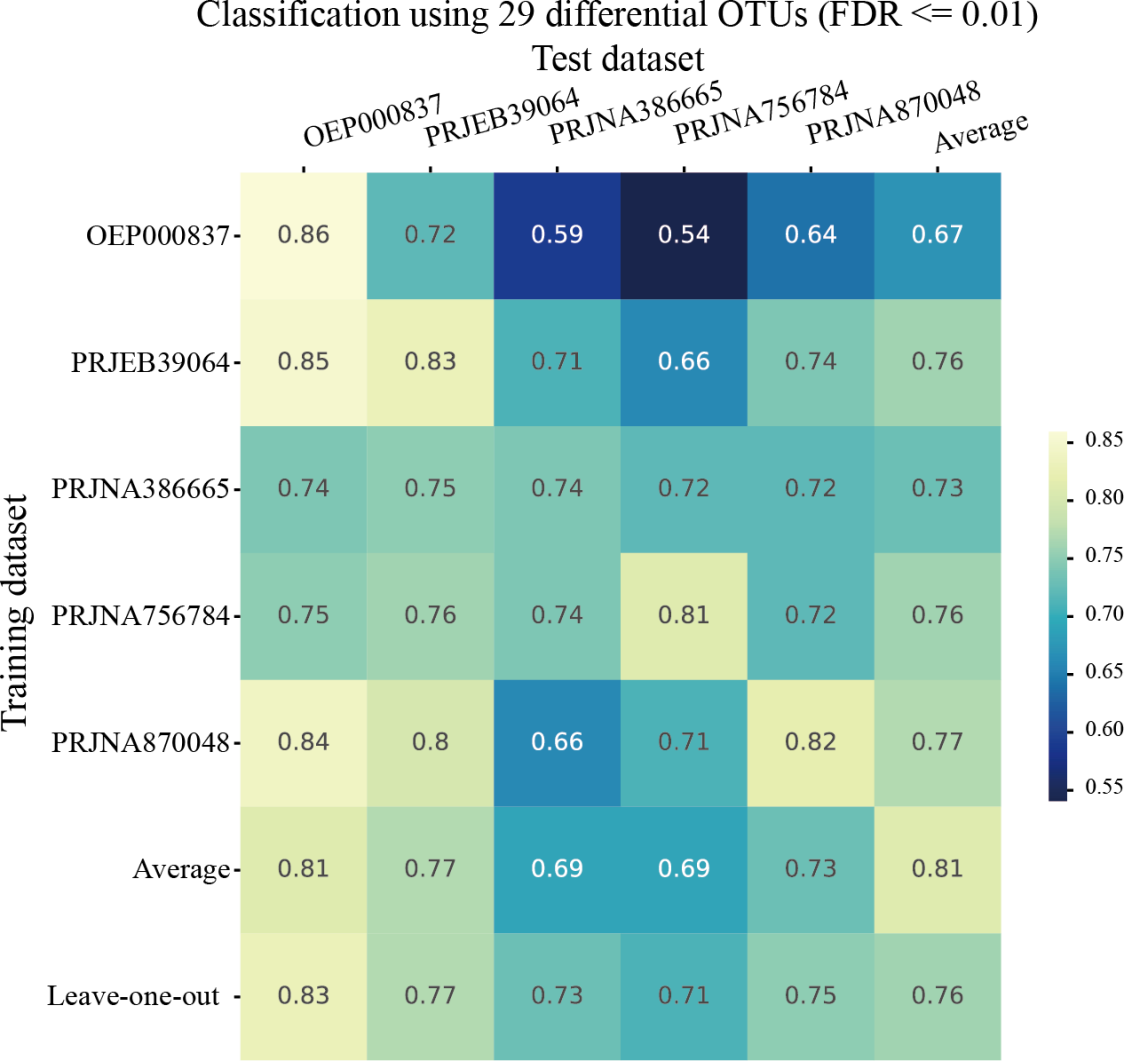
Performance of discriminating OSCC samples from healthy controls using 29 differential features. The heatmap depicts AUC scores from RF classifiers. The diagonal elements represent the cross-validation performance within each individual data set. The off-diagonal elements denote study-to-study predictions, with the training data sets along the rows and the test data sets along the columns. The “average”’ row shows the mean values for each column, with the last entry being the overall average of the diagonal values. The “leave-one-out” row displays the AUCs obtained by training the classifier with all data sets except the data set in the corresponding column and predicting the disease classes for the left-out data set. The “average” column provides the mean AUC values across each row.

To evaluate the potential of these differentially abundant microbes as global microbial biomarkers, we constructed random forest classification models using their abundance profiles to discriminate between OSCC and healthy samples. The results demonstrated that the AUCs for within-study cross-validation ranged from 0.74 to 0.86, with an average of 0.81 (Fig. 4). Cross-study prediction accuracies spanned from 0.54 to 0.85, which were lower than cross-validation performance, aligning with the variability between studies. Study OEP000837 exhibited relatively poorer predictive performance on other data sets, potentially due to its smallest sample size (Table 1). The leave-one-study-out model consistently performed equal to or better than the average of individual data set-based RF models, suggesting that integrating information from multiple data sets enhances model robustness by capturing broader biological variability and mitigating study-specific biases and highlighting the advantage of leveraging diverse data sets for universal biomarkers. Taken together, the results provide strong support for their potential as global microbial biomarkers for OSCC, warranting further validation and investigation into their functional roles in disease pathogenesis.

### Age-associated microbial shifts in OSCC patients

**Fig 5:**
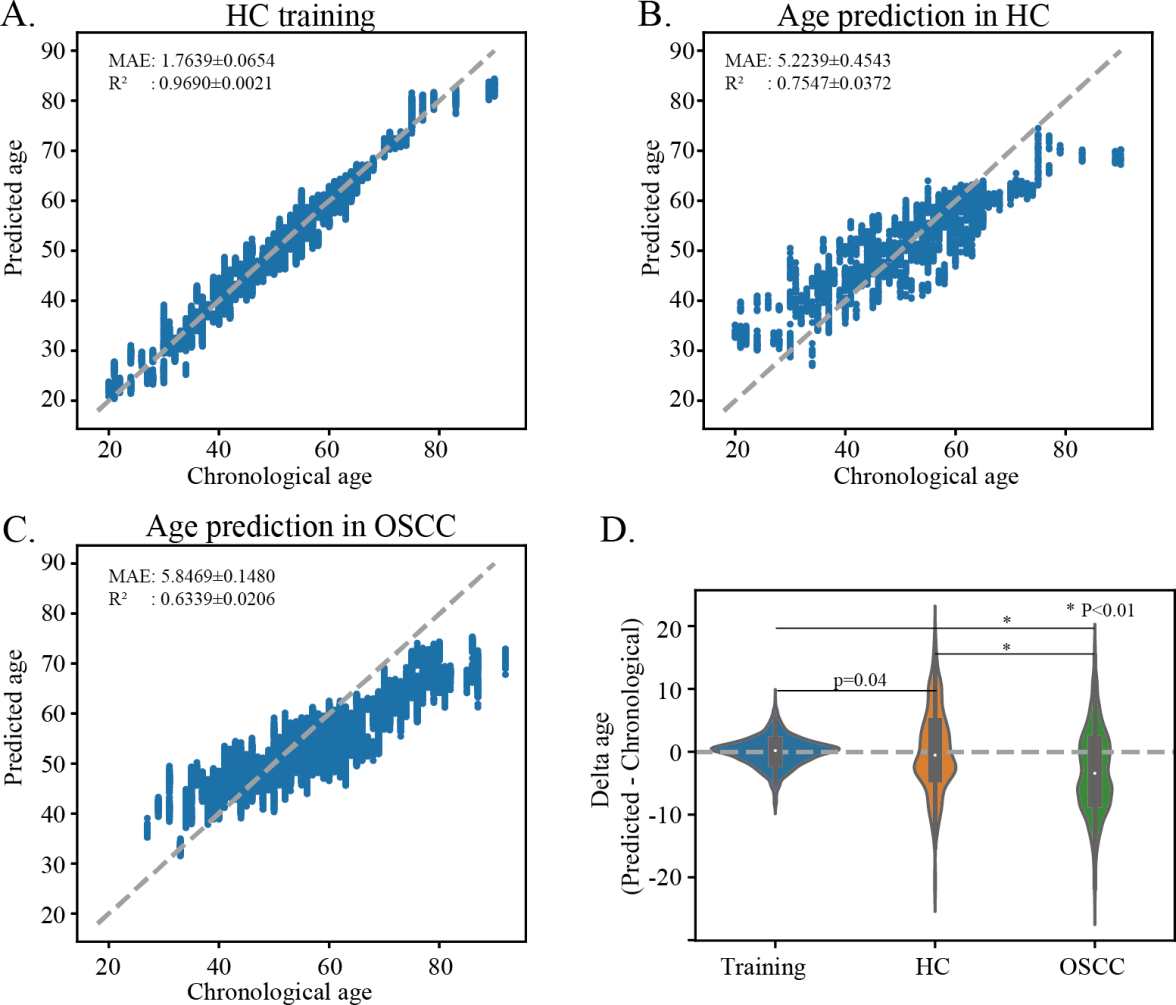
Age-related oral microbiome shifts in OSCC. (**A–C**) The RF model was trained on the healthy oral microbiome to predict host microbial age. The average MAEs were 1.76, 5.14, and 5.82 for the healthy training set, healthy cross-validation set, and OSCC set, respectively. (**D**) The delta between predicted and chronological ages in healthy training, healthy cross-validation, and OSCC samples.

The composition of the oral microbiome gradually changes with aging in healthy individuals (31, 51). Research has suggested that oral diseases may perturb this process (52). To explore how OSCC impacts age-related shifts in the oral microbiome, we pooled all microbial samples with age information from included data sets. A regression model was trained on healthy subject data to predict the salivary microbiome age of OSCC samples. This salivary microbiome aging model yielded a mean absolute error of 5.23 ± 0.45 years (Fig. 5B) for cross-validation within the healthy individuals, consistent with our previous results (31). However, when the model was applied to OSCC patients, the MAE increased to 5.84 ± 0.15 years (Fig. 5C), indicating that age-related microbiome signatures from healthy individuals cannot accurately translate to the OSCC population. Specifically, the salivary microbiome-predicted age was significantly lower than the chronological age for OSCC compared to healthy individuals (Fig. 5D), suggesting that OSCC may impede the normal aging of the salivary microbiome, which warrants further mechanistic investigations into dysbiotic microbiome aging in disease pathogenesis.

### Gender-associated microbial shifts in OSCC patients

**Fig 6:**
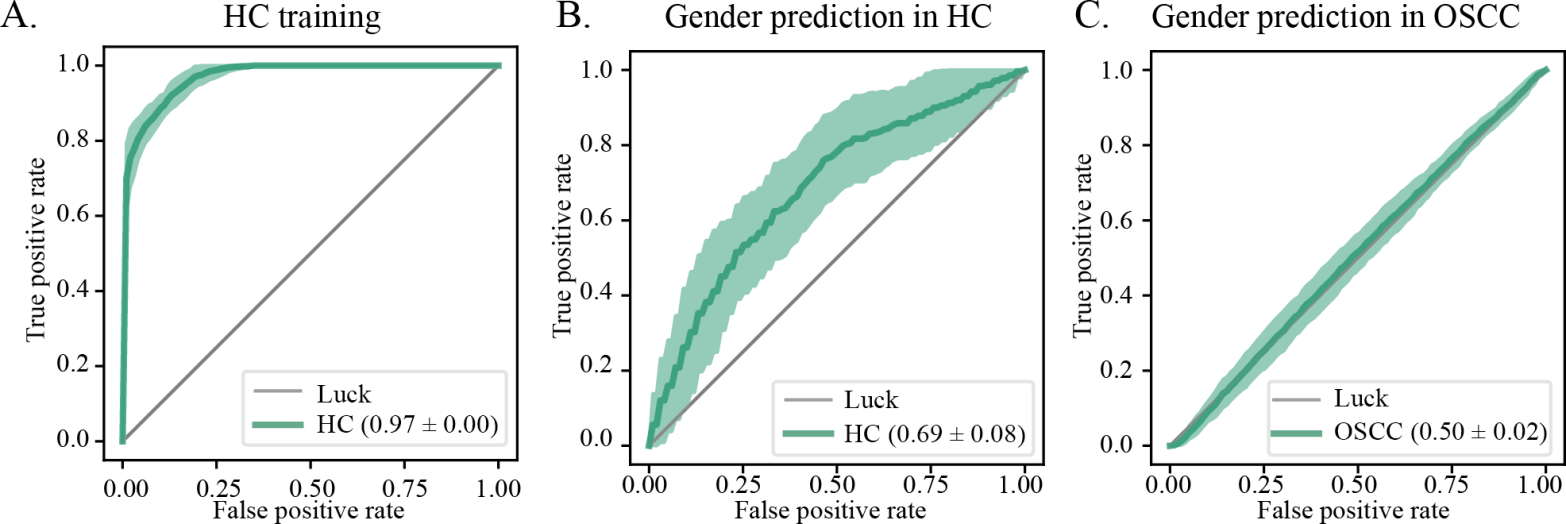
Gender-related oral microbiome shifts in OSCC. (**A–C**) The RF model was trained on healthy oral microbiome to classify host gender. The average AUCs were 0.97, 0.71, and 0.5 for the healthy training set, healthy cross-validation set, and OSCC set, respectively.

We also investigated the influence of gender on the oral microbiome. Similarly, we incorporated samples with gender information from multiple data sets and trained the microbiome data from healthy individuals to predict the gender of OSCC subjects. The AUC of cross-validation within healthy samples was 0.71 (Fig. 6B); however, it decreased to 0.51 when applied to OSCC samples (Fig. 6C), suggesting that although the salivary microbiome composition reflects host gender information in healthy individuals to some degree, these gender-associated microbial features cannot be effectively generalized to OSCC patients. It is plausible that the disease-associated microenvironmental perturbations may remodel the microbial landscape, diminishing or disrupting the associations between host characteristics and the microbiome, thereby limiting the applicability of machine learning models based on healthy microbiome profiles in disease contexts.

## DISCUSSION

We conducted a systematic search and screening of the literature related to OSCC salivary microbiome published between January 2010 and February 2023, ultimately retaining 11 studies with accessible metadata and 16S amplicon sequencing raw data for meta-analysis. Unified analytical frameworks were employed to remove variations introduced by different data processing methods. Batch effects are a key impediment to meta-analysis, thus the newly developed ConQuR algorithm, specifically tailored for microbiome data, was utilized to mitigate batch effects. Adonis tests revealed significant differences in microbial community composition between healthy individuals and OSCC patients. Random effects models demonstrated a significant increase in the Shannon index and Faith’s PD in OSCC patients compared to healthy controls. Furthermore, we identified cross-study OSCC-associated microbial markers, and these microbes could accurately distinguish OSCC and healthy samples both within and across data sets. Notably, we discovered a significant perturbation in the microbial signatures that are typically associated with age and gender within the OSCC patients.

By integrating 11 data sets, we first evaluated the impact of various factors on the saliva microbiome, including study, sequencing region, sample type, and disease status. Other factors, such as tumor sites, TNM stages, smoking, and drinking habits, were not included due to varying degrees of missing data across different data sets. Our results revealed that inter-study variation was the most substantial contributor to the variation observed in the oral microbiome, in line with other research (53, 54). The ConQuR algorithm is characterized by its ability to correct batch effects while preserving biologically relevant signals of interest. After applying ConQuR, we observed a considerable overlap in the centroids across different data sets (Fig. 2B), indicating effective control of inter-study heterogeneity. Furthermore, the Adonis test showed a relatively minor change in the *R*^2^ value for disease status compared to the substantial reduction in *R*^2^ for other factors. These results demonstrate the utility of ConQuR in deconvoluting batch effects from biologically relevant variation in microbiome studies.

Random effects models are a well-established approach in meta-analysis, as they prudently account for stochastic variability across constituent studies, thereby fortifying the generalizability and robustness of the cumulative findings (41, 55, 56). The combined Shannon index and Faith’s PD were significantly higher in OSCC compared to healthy samples, while there was no significant difference in observed OTUs between the two groups. We observed that this could be attributed to the substantially higher observed OTUs in healthy individuals compared to OSCC patients in studies PRJNA756784 and PRJNA751046, consequently leading to a non-significant difference in the combined results. Notably, these two studies were Southern Asian cohorts, focusing on long-term tobacco chewing, with the healthy groups including tobacco chewers. Research has demonstrated that smoking can influence oral microbiome diversity (57). Similarly, the use of smokeless tobacco products in Southern Asia, which often involves the chewing of tobacco leaves, spices, and oral mucosa-stimulating ingredients, has been shown to increase salivary microbiota alpha diversity in non-OSCC individuals (13, 15). This may explain the higher OTU counts and Faith’s PD observed in studies PRJNA756784 and PRJNA751046. However, as only limited data sets contained information on smoking status, we were unable to comprehensively evaluate the impact of this factor. Overall, by combining eight data sets, we found that the Shannon index and Faith’s PD of the salivary microbiome were significantly elevated in OSCC patients compared to healthy individuals.

Adonis analyses based on different beta-diversity metrics consistently revealed distinct salivary microbiome profiles between OSCC patients and healthy individuals across studies, suggesting that the salivary microbiome in OSCC patients is significantly perturbed across diverse population cohorts. Through random effects models, we identified cross-data set microbial signatures associated with OSCC. The bacteria enriched in OSCC samples included *Streptococcus*, *Lactobacillus*, *Prevotella*, *Bulleidia moorei*, *Haemophilus*, etc. *Streptococcus* was the most frequent genera in the saliva of oral cancer patients with active lesions (16), and it was reported to produce carcinogen acetaldehyde from ethanol and glucose metabolism (58, 59). *Lactobacillus* has been found to be more abundant in the saliva microbiota of OSCC patients compared to control samples (12, 13), particularly in patients with advanced T and N stages (60). Frank et al. (61) proposed that the over-enrichment of *Lactobacillus* activates the aryl hydrocarbon receptor (AhR) pathway, leading to the inhibition of immunity and the progression of OSCC. Conversely, bacteria enriched in control samples included *Actinomyces*, *Corynebacterium*, CW040, *Prevotella,* and *Leptotrichia. Actinomyces* spp. are generally recognized for their low virulence and are considered to be part of the normal microbiota found in the oral cavity (62). In the study conducted by Chen et al. (14), *Actinomyces* sp. HMT-180 was identified as a core species with a notable abundance within the normal cohort. *Corynebacterium* was listed as the predominant commensal taxa in healthy saliva microbiota (63), and its higher abundance was associated with a lower risk of head and neck cancer (64). The abundance of *Leptotrichia* decreased with oral mucositis progression (65), and a higher abundance of intratumoral *Leptotrichia* was related to a higher survival rate (66).

Furthermore, we found that random forest models constructed from these differential microbial features could effectively predict host disease status. Notably, incorporating more data sets for training further enhanced the performance of the classification models. These results not only confirm the role of these microbes as OSCC markers but also underscore the importance of meta-analysis, as integrating a larger body of research can yield more robust and generalizable findings. The consistent identification of OSCC-associated microbial dysbiosis across diverse cohorts highlights the potential utility of the salivary microbiome as a non-invasive biomarker for OSCC detection. Mechanistic research is warranted to investigate the causal relationship between dysregulated microbiome and OSCC development.

Considering the unknown correlation between agng and the oral microbiome, we trained an RF regression model on healthy human oral microbiome for age prediction. The mean MAE on the test data was 5.22 ± 0.75 years, slightly higher than the results in our previous study (MAE = 4.5 ± 0.14 years) (31), possibly due to the larger sample size in the previous study, which yielded a more accurate estimate. Interestingly, when the model was applied to OSCC samples, oral microbiome age appeared to lag behind chronological age, suggesting the disruption of normal aging patterns of the oral microbiome in OSCC carcinogenesis. This contrasts with a previous study reporting “accelerated” oral microbiome aging in gingivitis (52). This discrepancy may suggest that different oral diseases may induce shifts in the oral microbiome in divergent directions.

Similarly, we examined the association between gender and the salivary microbiome. The AUC of 0.71 in healthy samples suggests detectable differences in oral microbiome composition between males and females in the healthy population, likely arising from factors such as hormonal differences, physiological variations, or behavioral and environmental influences that shape the microbiome (32, 33). However, when applying these gender prediction models to OSCC samples, the AUC dropped significantly to 0.51, indicating no better performance than random guessing. This stark contrast suggests that the gender-specific microbiome patterns observed in healthy states were disrupted or diminished in the context of oral squamous cell carcinoma. The alteration of age and gender specificity in the salivary microbiome implies a potential decoupling of host-microbiome interactions, which may have implications for disease etiology, progression, and therapeutic strategies. Further studies are needed to elucidate the underlying biological processes driving these complex microbiome perturbations in OSCC.

Compared to existing meta-analyses on OSCC (67–69), this study introduces several improvements. The literature has demonstrated differences in the microbiome among tissue tumors, saliva, and other oral surfaces (22). Given the non-invasive nature and ease of collection, saliva serves as an ideal medium for routine screening and early diagnosis. Our study focused exclusively on the saliva microbiome, yielding more precise microbial conclusions. We employed state-of-the-art bioinformatics methods to uniformly process sequencing data across studies, which allows us to observe consistent microbial changes, enhancing the reliability and comparability of our findings. Additionally, our analysis revealed age- and gender-related microbiome dysbiosis in OSCC patients, potentially informing more personalized diagnostic and therapeutic strategies.

Our study has limitations. First, due to the lack of comprehensive clinical data in the data sets, we were unable to fully evaluate the impact of potential confounding factors, such as age, gender, and smoking status, on the oral microbiome. Complete cohort information would facilitate a deep exploration of the associations between OSCC, host characteristics, and lifestyle factors. Second, this study was based on 16S amplicon sequencing data, thus we focused on changes in microbial community composition. The functional alterations in the microbiome and the underlying mechanisms in OSCC remained to be elucidated. Integrating multi-omics data, such as metagenomics, meta-transcriptomics, and metabolomics, would aid in comprehensively understanding the functional dysbiosis associated with OSCC (70). Third, although we established an association between OSCC and salivary microbiome dysbiosis, the causal relationship remains unclear, which warrants longitudinal cohort studies and mechanistic investigations. Fourth, tumor tissue samples and swab samples from specific oral lesions were intentionally excluded from our study due to the acknowledged heterogeneity in the microbiome profiles they may introduce. Further data are needed to comprehensively assess the difference and generalizability of these samples compared to the salivary microbiome.

In conclusion, by integrating salivary microbiome data from 11 studies, this meta-analysis identified consistent and significant microbial differences between healthy and OSCC groups. Cross-data set microbial diagnostic signatures were identified and could be used to classify OSCC patients from healthy individuals. We also unveiled disruptions in the age- and gender-associated salivary microbiome signatures in OSCC, suggesting that OSCC may alter the normal host-microbiome interactions. Our work provides insights into the salivary microbiome dysbiosis in OSCC and suggests the potential of the salivary microbiota for early detection in high-risk OSCC populations.

## Data Availability

The raw data and metadata of studies incorporated in our meta-analysis are listed in Table 1 and are available from public databases or from the authors upon request. The analysis code is available at https://github.com/zxlab/OSCC-meta-analysis.git.

## SUPPLEMENTAL MATERIAL

Supplemental material msystems.01247-24-s0001.pdf Supplemental table and figures.

ASM does not own the copyrights to Supplemental Material that may be linked to, or accessed through, an article. The authors have granted ASM a non-exclusive, world-wide license to publish the Supplemental Material files. Please contact the corresponding author directly for reuse.

## References

[1] Hu Y, Amir A, Huang X, Li Y, Huang S, Wolfe E, Weiss S, Knight R, Xu ZZ. 2022. Diurnal and eating-associated microbial patterns revealed via high-frequency saliva sampling. Genome Res 32:1112–1123. doi:10.1101/gr.276482.121 35688483 PMC9248889

[2] Baker JL, Mark Welch JL, Kauffman KM, McLean JS, He X. 2024. The oral microbiome: diversity, biogeography and human health. Nat Rev Microbiol 22:89–104. doi:10.1038/s41579-023-00963-6 37700024 PMC11084736

[3] Hou K, Wu Z-X, Chen X-Y, Wang J-Q, Zhang D, Xiao C, Zhu D, Koya JB, Wei L, Li J, Chen Z-S. 2022. Microbiota in health and diseases. Sig Transduct Target Ther 7:1–28. doi:10.1038/s41392-022-00974-4 PMC9034083 35461318

[4] Li X, Liu Y, Yang X, Li C, Song Z. 2022. The oral microbiota: community composition, influencing factors, pathogenesis, and interventions. Front Microbiol 13:895537–895555. doi:10.3389/fmicb.2022.895537 35572634 PMC9100676

[5] Tan Y, Wang Z, Xu M, Li B, Huang Z, Qin S, Nice EC, Tang J, Huang C. 2023. Oral squamous cell carcinomas: state of the field and emerging directions. Int J Oral Sci 15:44. doi:10.1038/s41368-023-00249-w 37736748 PMC10517027

[6] Sung H, Ferlay J, Siegel RL, Laversanne M, Soerjomataram I, Jemal A, Bray F. 2021. Global cancer statistics 2020: GLOBOCAN estimates of incidence and mortality worldwide for 36 cancers in 185 countries. CA A Cancer J Clinicians 71:209–249. doi:10.3322/caac.21660 33538338

[7] Swaminathan D, George NA, Thomas S, Iype EM. 2024. Factors associated with delay in diagnosis of oral cancers. Cancer Treat Res Commun 40:100831. doi:10.1016/j.ctarc.2024.100831 38996584

[8] Sousa‐Neto SS, Martins AFL, Moreira VHL de O, Pereira JGB, Freitas NMA, Curado MP, Leles CR, Mendonça EF. 2024. The association between referral by specialists in oral diagnosis on survival rates of patients with oral cancer: a retrospective cohort study. J Oral Pathol Med 53:358–365. doi:10.1111/jop.13546 38745372

[9] González-Ruiz I, Ramos-García P, Ruiz-Ávila I, González-Moles MÁ. 2023. Early diagnosis of oral cancer: a complex polyhedral problem with a difficult solution. Cancers (Basel) 15:1–24. doi:10.3390/cancers15133270 PMC10340032 37444379

[10] Stashenko P, Yost S, Choi Y, Danciu T, Chen T, Yoganathan S, Kressirer C, Ruiz-Tourrella M, Das B, Kokaras A, Frias-Lopez J. 2019. The oral mouse microbiome promotes tumorigenesis in oral squamous cell carcinoma. mSystems 4:e00323-19. doi:10.1128/mSystems.00323-19 PMC6687944 31387932

[11] Lafuente Ibáñez de Mendoza I, Maritxalar Mendia X, García de la Fuente AM, Quindós Andrés G, Aguirre Urizar JM. 2020. Role of Porphyromonas gingivalis in oral squamous cell carcinoma development: a systematic review. J Periodont Res 55:13–22. doi:10.1111/jre.12691 31529626

[12] Zhu H, Yip HC, Cheung MK, Chan HC, Ng C, Lau EHL, Yeung ZWC, Wong EWY, Leung L, Qu X, Wang D, Cai L, Chan PKS, Chan JYK, Chen Z. 2023. Convergent dysbiosis of upper aerodigestive microbiota between patients with esophageal and oral cavity squamous cell carcinoma. Int J Cancer 152:1903–1915. doi:10.1002/ijc.34460 36752573

[13] Srivastava A, Mishra S, Garg PK, Dubey AK, Deo SVS, Verma D. 2022. Comparative and analytical characterization of the oral bacteriome of smokeless tobacco users with oral squamous cell carcinoma. Appl Microbiol Biotechnol 106:4115–4128. doi:10.1007/s00253-022-11980-5 35596785

[14] Chen J-W, Wu J-H, Chiang W-F, Chen Y-L, Wu W-S, Wu L-W. 2021. Taxonomic and functional dysregulation in salivary microbiomes during oral carcinogenesis. Front Cell Infect Microbiol 11:663068. doi:10.3389/fcimb.2021.663068 34604102 PMC8482814

[15] Sawant S, Dugad J, Parikh D, Srinivasan S, Singh H. 2021. Identification & correlation of bacterial diversity in oral cancer and long-term tobacco chewers- a case-control pilot study. J Med Microbiol 70:1–11. doi:10.1099/jmm.0.001417 34553683

[16] Granato DC, Neves LX, Trino LD, Carnielli CM, Lopes AFB, Yokoo S, Pauletti BA, Domingues RR, Sá JO, Persinoti G, Paixão DAA, Rivera C, de Sá Patroni FM, Tommazetto G, Santos-Silva AR, Lopes MA, de Castro G Jr, Brandão TB, Prado-Ribeiro AC, Squina FM, Telles GP, Paes Leme AF. 2021. Meta-omics analysis indicates the saliva microbiome and its proteins associated with the prognosis of oral cancer patients. Biochim Biophysica Acta (BBA) Proteins Proteom 1869:140659. doi:10.1016/j.bbapap.2021.140659 33839314

[17] Yang K, Wang Y, Zhang S, Zhang D, Hu L, Zhao T, Zheng H. 2021. Oral microbiota analysis of tissue pairs and saliva samples from patients with oral squamous cell carcinoma - a pilot study. Front Microbiol 12:719601. doi:10.3389/fmicb.2021.719601 34712209 PMC8546327

[18] Chan JYK, Ng CWK, Lan L, Fung S, Li J-W, Cai L, Lei P, Mou Q, Meehan K, Lau EHL, Yeung Z, Chan KCA, Wong EWY, Chan PKS, Chen Z. 2021. Restoration of the oral microbiota after surgery for head and neck squamous cell carcinoma is associated with patient outcomes. Front Oncol 11:737843. doi:10.3389/fonc.2021.737843 34692514 PMC8527003

[19] Vesty A, Gear K, Biswas K, Radcliff FJ, Taylor MW, Douglas RG. 2018. Microbial and inflammatory-based salivary biomarkers of head and neck squamous cell carcinoma. Clin Exp Dent Res 4:255–262. doi:10.1002/cre2.139 30603107 PMC6305924

[20] Lee W-H, Chen H-M, Yang S-F, Liang C, Peng C-Y, Lin F-M, Tsai L-L, Wu B-C, Hsin C-H, Chuang C-Y, Yang T, Yang T-L, Ho S-Y, Chen W-L, Ueng K-C, Huang H-D, Huang C-N, Jong Y-J. 2017. Bacterial alterations in salivary microbiota and their association in oral cancer. Sci Rep 7:16540. doi:10.1038/s41598-017-16418-x 29184122 PMC5705712

[21] Yang C-Y, Yeh Y-M, Yu H-Y, Chin C-Y, Hsu C-W, Liu H, Huang P-J, Hu S-N, Liao C-T, Chang K-P, Chang Y-L. 2018. Oral microbiota community dynamics associated with oral squamous cell carcinoma staging. Front Microbiol 9:862. doi:10.3389/fmicb.2018.00862 29774014 PMC5943489

[22] Zhou X, Hao Y, Peng X, Li B, Han Q, Ren B, Li M, Li L, Li Y, Cheng G, Li J, Ma Y, Zhou X, Cheng L. 2021. The clinical potential of oral microbiota as a screening tool for oral squamous cell carcinomas. Front Cell Infect Microbiol 11:728933. doi:10.3389/fcimb.2021.728933 34485181 PMC8416267

[23] Heng W, Wang W, Dai T, Jiang P, Lu Y, Li R, Zhang M, Xie R, Zhou Y, Zhao M, Duan N, Ye Z, Yan F, Wang X. 2022. Oral bacteriome and mycobiome across stages of oral carcinogenesis. Microbiol Spectr 10:e02737-22. doi:10.1128/spectrum.02737-22 36445134 PMC9769585

[24] Lan QY, Zhang C, Hua H, Hu XS. 2023. Compositional and functional changes in the salivary microbiota related to oral leukoplakia and oral squamous cell carcinoma: a case control study. BMC Oral Health 23:1021. doi:10.1186/s12903-023-03760-y 38115005 PMC10731685

[25] Mäkinen AI, Pappalardo VY, Buijs MJ, Brandt BW, Mäkitie AA, Meurman JH, Zaura E. 2023. Salivary microbiome profiles of oral cancer patients analyzed before and after treatment. Microbiome 11:171. doi:10.1186/s40168-023-01613-y 37542310 PMC10403937

[26] Kaan AMM, Kahharova D, Zaura E. 2021. Acquisition and establishment of the oral microbiota. Periodontol 2000 86:123–141. doi:10.1111/prd.12366 33690935 PMC8252790

[27] Xu H, Tian B, Shi W, Tian J, Wang W, Qin M. 2022. Maturation of the oral microbiota during primary teeth eruption: a longitudinal, preliminary study. J Oral Microbiol 14:2051352. doi:10.1080/20002297.2022.2051352 35309409 PMC8933015

[28] Burcham ZM, Garneau NL, Comstock SS, Tucker RM, Knight R, Metcalf JL, Miranda A, Reinhart B, Meyers D, Woltkamp D, Boxer E, Hutchens J, Kim K, Archer M, McAteer M, Huss P, Defonseka R, Stahle S, Babu S, Nuessle T, Schowinsky V, Covert W, Truman W, Reusser W. 2020. Patterns of oral microbiota diversity in adults and children: a crowdsourced population study. Sci Rep 10:2133. doi:10.1038/s41598-020-59016-0 32034250 PMC7005749

[29] Saadaoui M, Singh P, Al Khodor S. 2021. Oral microbiome and pregnancy: a bidirectional relationship. J Reprod Immunol 145:103293. doi:10.1016/j.jri.2021.103293 33676065

[30] Liu S, Wang Y, Zhao L, Sun X, Feng Q. 2020. Microbiome succession with increasing age in three oral sites. Aging (Milano) 12:7874–7907. doi:10.18632/aging.103108 PMC7244077 32379704

[31] Huang S, Haiminen N, Carrieri A-P, Hu R, Jiang L, Parida L, Russell B, Allaband C, Zarrinpar A, Vázquez-Baeza Y, Belda-Ferre P, Zhou H, Kim H-C, Swafford AD, Knight R, Xu ZZ. 2020. Human skin, oral, and gut microbiomes predict chronological age. mSystems 5:e00630-19. doi:10.1128/mSystems.00630-19 32047061 PMC7018528

[32] Liu X, Tong X, Jie Z, Zhu J, Tian L, Sun Q, Ju Y, Zou L, Lu H, Qiu X, et al.. 2023. Sex differences in the oral microbiome, host traits, and their causal relationships. i Sci 26:105839. doi:10.1016/j.isci.2022.105839 PMC9843272 36660475

[33] Ma ZS, Li W. 2019. How and why men and women differ in their microbiomes: medical ecology and network analyses of the microgenderome. Adv Sci (Weinh) 6:1902054. doi:10.1002/advs.201902054 31832327 PMC6891928

[34] Zeng B, Tan J, Guo G, Li Z, Yang L, Lao X, Wang D, Ma J, Zhang S, Liao G, Liang Y. 2022. The oral cancer microbiome contains tumor space-specific and clinicopathology-specific bacteria. Front Cell Infect Microbiol 12:942328. doi:10.3389/fcimb.2022.942328 36636719 PMC9831678

[35] Chen MY, Chen JW, Wu LW, Huang KC, Chen JY, Wu WS, Chiang WF, Shih CJ, Tsai KN, Hsieh WT, Ho YH, Wong TY, Wu JH, Chen YL. 2021. Carcinogenesis of male oral submucous fibrosis alters salivary microbiomes. J Dent Res 100:397–405. doi:10.1177/0022034520968750 33089709

[36] Bolyen E, Rideout JR, Dillon MR, Bokulich NA, Abnet CC, Al-Ghalith GA, Alexander H, Alm EJ, Arumugam M, Asnicar F, et al.. 2019. Reproducible, interactive, scalable and extensible microbiome data science using QIIME 2. Nat Biotechnol 37:852–857. doi:10.1038/s41587-019-0209-9 31341288 PMC7015180

[37] Amir A, McDonald D, Navas-Molina JA, Kopylova E, Morton JT, Zech Xu Z, Kightley EP, Thompson LR, Hyde ER, Gonzalez A, Knight R. 2017. Deblur rapidly resolves single-nucleotide community sequence patterns. mSystems 2:e00191-16. doi:10.1128/mSystems.00191-16 28289731 PMC5340863

[38] DeSantis TZ, Hugenholtz P, Larsen N, Rojas M, Brodie EL, Keller K, Huber T, Dalevi D, Hu P, Andersen GL. 2006. Greengenes, a chimera-checked 16S rRNA gene database and workbench compatible with ARB. Appl Environ Microbiol 72:5069–5072. doi:10.1128/AEM.03006-05 16820507 PMC1489311

[39] Ling W, Lu J, Zhao N, Lulla A, Plantinga AM, Fu W, Zhang A, Liu H, Song H, Li Z, Chen J, Randolph TW, Koay WLA, White JR, Launer LJ, Fodor AA, Meyer KA, Wu MC. 2022. Batch effects removal for microbiome data via conditional quantile regression. Nat Commun 13:1–14. doi:10.1038/s41467-022-33071-9 36109499 PMC9477887

[40] Bisanz JE, Upadhyay V, Turnbaugh JA, Ly K, Turnbaugh PJ. 2019. Meta-analysis reveals reproducible gut microbiome alterations in response to a high-fat diet. Cell Host Microbe 26:265–272. doi:10.1016/j.chom.2019.06.013 31324413 PMC6708278

[41] Shah MS, DeSantis TZ, Weinmaier T, McMurdie PJ, Cope JL, Altrichter A, Yamal JM, Hollister EB. 2018. Leveraging sequence-based faecal microbial community survey data to identify a composite biomarker for colorectal cancer. Gut 67:882–891. doi:10.1136/gutjnl-2016-313189 28341746

[42] Anders S, Huber W. 2010. Differential expression analysis for sequence count data. Genome Biol 11:1–12. doi:10.1186/gb-2010-11-10-r106 PMC3218662 20979621

[43] Viechtbauer W. 2010. Conducting meta-analyses in R with the metafor package. J Stat Softw 36:1–48. doi:10.18637/jss.v036.i03

[44] Pedregosa F, Varoquaux G, Gramfort A, Michel V, Thirion B, Grisel O, Blondel M, Prettenhofer P, Weiss R, Dubourg V, Vanderplas J, Passos A, Cournapeau D, Brucher M, Perrot M, Duchesnay E. 2011. Scikit-learn: machine learning in Python. J Mach Learn Res 12:2825–2830. doi:10.5555/1953048.2078195

[45] Pasolli E, Truong DT, Malik F, Waldron L, Segata N. 2016. Machine learning meta-analysis of large metagenomic datasets: tools and biological insights. PLoS Comput Biol 12:e1004977. doi:10.1371/journal.pcbi.1004977 27400279 PMC4939962

[46] Ye E, Sun H, Leone MJ, Paixao L, Thomas RJ, Lam AD, Westover MB. 2020. Association of sleep electroencephalography-based brain age index with dementia. JAMA Netw Open 3:e2017357. doi:10.1001/jamanetworkopen.2020.17357 32986106 PMC7522697

[47] Jo R, Nishimoto Y, Umezawa K, Yama K, Aita Y, Ichiba Y, Murakami S, Kakizawa Y, Kumagai T, Yamada T, Fukuda S. 2019. Comparison of oral microbiome profiles in stimulated and unstimulated saliva, tongue, and mouth-rinsed water. Sci Rep 9:1–7. doi:10.1038/s41598-019-52445-6 31695050 PMC6834574

[48] Fan X, Peters BA, Min D, Ahn J, Hayes RB. 2018. Comparison of the oral microbiome in mouthwash and whole saliva samples. PLoS One 13:e0194729. doi:10.1371/journal.pone.0194729 29641531 PMC5894969

[49] Omori M, Kato-Kogoe N, Sakaguchi S, Fukui N, Yamamoto K, Nakajima Y, Inoue K, Nakano H, Motooka D, Nakano T, Nakamura S, Ueno T. 2021. Comparative evaluation of microbial profiles of oral samples obtained at different collection time points and using different methods. Clin Oral Investig 25:2779–2789. doi:10.1007/s00784-020-03592-y 32975702

[50] Caselli E, Fabbri C, D’Accolti M, Soffritti I, Bassi C, Mazzacane S, Franchi M. 2020. Defining the oral microbiome by whole-genome sequencing and resistome analysis: the complexity of the healthy picture. BMC Microbiol 20:120. doi:10.1186/s12866-020-01801-y 32423437 PMC7236360

[51] Willis JR, Saus E, Iraola-Guzmán S, Ksiezopolska E, Cozzuto L, Bejarano LA, Andreu-Somavilla N, Alloza-Trabado M, Blanco A, Puig-Sola A, Broglio E, Carolis C, Ponomarenko J, Hecht J, Gabaldón T. 2022. Citizen-science reveals changes in the oral microbiome in Spain through age and lifestyle factors. NPJ Biofilms Microbiomes 8:38. doi:10.1038/s41522-022-00279-y 35585074 PMC9117221

[52] Huang S, He T, Yue F, Xu X, Wang L, Zhu P, Teng F, Sun Z, Liu X, Jing G, Su X, Jin L, Liu J, Xu J. 2021. Longitudinal multi-omics and microbiome meta-analysis identify an asymptomatic gingival state that links gingivitis, periodontitis, and aging. mBio 12:e03281-20. doi:10.1128/mBio.03281-20 33688007 PMC8092283

[53] Li Y, Hu YC, Zhan X, Song Y, Xu M, Wang SJ, Huang XC, Xu ZZ. 2023. Meta-analysis reveals Helicobacter pylori mutual exclusivity and reproducible gastric microbiome alterations during gastric carcinoma progression. Gut Microbes 15:2197835. doi:10.1080/19490976.2023.2197835 37020297 PMC10078126

[54] Wirbel J, Pyl PT, Kartal E, Zych K, Kashani A, Milanese A, Fleck JS, Voigt AY, Palleja A, Ponnudurai R, et al.. 2019. Meta-analysis of fecal metagenomes reveals global microbial signatures that are specific for colorectal cancer. Nat Med 25:679–689. doi:10.1038/s41591-019-0406-6 30936547 PMC7984229

[55] Thomas AM, Manghi P, Asnicar F, Pasolli E, Armanini F, Zolfo M, Beghini F, Manara S, Karcher N, Pozzi C, et al.. 2019. Metagenomic analysis of colorectal cancer datasets identifies cross-cohort microbial diagnostic signatures and a link with choline degradation. Nat Med 25:667–678. doi:10.1038/s41591-019-0405-7 30936548 PMC9533319

[56] Dettori JR, Norvell DC, Chapman JR. 2022. Fixed-effect vs random-effects models for meta-analysis: 3 points to consider. Global Spine J 12:1624–1626. doi:10.1177/21925682221110527 35723546 PMC9393987

[57] Wang X, Mi Q, Yang J, Guan Y, Zeng W, Xiang H, Liu X, Yang W, Yang G, Li X, Cui Y, Gao Q. 2022. Effect of electronic cigarette and tobacco smoking on the human saliva microbial community. Braz J Microbiol 53:991–1000. doi:10.1007/s42770-022-00721-5 35229279 PMC9151971

[58] Moritani K, Takeshita T, Shibata Y, Ninomiya T, Kiyohara Y, Yamashita Y. 2015. Acetaldehyde production by major oral microbes. Oral Dis 21:748–754. doi:10.1111/odi.12341 25809116

[59] Tagaino R, Washio J, Abiko Y, Tanda N, Sasaki K, Takahashi N. 2019. Metabolic property of acetaldehyde production from ethanol and glucose by oral Streptococcus and Neisseria. Sci Rep 9:10446. doi:10.1038/s41598-019-46790-9 31320675 PMC6639336

[60] Guerrero-Preston R, White JR, Godoy-Vitorino F, Rodríguez-Hilario A, Navarro K, González H, Michailidi C, Jedlicka A, Canapp S, Bondy J, Dziedzic A, Mora-Lagos B, Rivera-Alvarez G, Ili-Gangas C, Brebi-Mieville P, Westra W, Koch W, Kang H, Marchionni L, Kim Y, Sidransky D. 2017. High-resolution microbiome profiling uncovers Fusobacterium nucleatum, Lactobacillus gasseri/johnsonii, and Lactobacillus vaginalis associated to oral and oropharyngeal cancer in saliva from HPV positive and HPV negative patients treated with surgery and chemo-radiation. Oncotarget 8:110931–110948. doi:10.18632/oncotarget.20677 29340028 PMC5762296

[61] Frank DN, Qiu Y, Cao Y, Zhang S, Lu L, Kofonow JM, Robertson CE, Liu Y, Wang H, Levens CL, Kuhn KA, Song J, Ramakrishnan VR, Lu S-L. 2022. A dysbiotic microbiome promotes head and neck squamous cell carcinoma. Oncogene 41:1269–1280. doi:10.1038/s41388-021-02137-1 35087236 PMC8882136

[62] Brook I. 2008. Anaerobic Gram-positive, nonsporulating bacilli (including actinomycosis), p 977–979. In Long SS (ed), Principles and practice of pediatric infectious disease, 3rd ed. W.B. Saunders, Edinburgh, Scotland.

[63] Torralba MG, Aleti G, Li W, Moncera KJ, Lin Y-H, Yu Y, Masternak MM, Golusinski W, Golusinski P, Lamperska K, Edlund A, Freire M, Nelson KE. 2021. Oral microbial species and virulence factors associated with oral squamous cell carcinoma. Microb Ecol 82:1030–1046. doi:10.1007/s00248-020-01596-5 33155101 PMC8551143

[64] Hayes RB, Ahn J, Fan X, Peters BA, Ma Y, Yang L, Agalliu I, Burk RD, Ganly I, Purdue MP, Freedman ND, Gapstur SM, Pei Z. 2018. Association of oral microbiome with risk for incident head and neck squamous cell cancer. JAMA Oncol 4:358–365. doi:10.1001/jamaoncol.2017.4777 29327043 PMC5885828

[65] Frey‐Furtado L, Magalhães I, Sampaio‐Maia B, Azevedo MJ. 2023. Oral microbiome characterization in oral mucositis patients—a systematic review. J Oral Pathology Medicine 52:911–918. doi:10.1111/jop.13492 37839408

[66] Hamada M, Inaba H, Nishiyama K, Yoshida S, Yura Y, Matsumoto-Nakano M, Uzawa N. 2023. Potential role of the intratumoral microbiota in prognosis of head and neck cancer. Int J Mol Sci 24:1–15. doi:10.3390/ijms242015456 PMC10607002 37895136

[67] Peter TK, Withanage MHH, Comnick CL, Pendleton C, Dabdoub S, Ganesan S, Drake D, Banas J, Xie XJ, Zeng E. 2022. Systematic review and meta-analysis of oral squamous cell carcinoma associated oral microbiome. Front Microbiol 13:968304. doi:10.3389/fmicb.2022.968304 36338051 PMC9632422

[68] Yu X, Shi Y, Yuan R, Chen Z, Dong Q, Han L, Wang L, Zhou J. 2023. Microbial dysbiosis in oral squamous cell carcinoma: a systematic review and meta-analysis. Heliyon 9:e13198. doi:10.1016/j.heliyon.2023.e13198 36793959 PMC9922960

[69] Delaney C, Veena CLR, Butcher MC, McLean W, Shaban SMA, Nile CJ, Ramage G. 2023. Limitations of using 16S rRNA microbiome sequencing to predict oral squamous cell carcinoma. APMIS 131:262–276. doi:10.1111/apm.13315 37002549

[70] Liu F, Lu H, Dong B, Huang X, Cheng H, Qu R, Hu Y, Zhong L, Guo Z, You Y, Xu ZZ. 2023. Systematic evaluation of the viable microbiome in the human oral and gut samples with spike-in Gram+/- bacteria. mSystems 8:e00738-22. doi:10.1128/msystems.00738-22 36971593 PMC10134872

