# Supplementary material for: Systematic analyses uncover robust salivary microbial signatures and host-microbiome perturbations in oral squamous cell carcinoma
Source: mSystems. 2025 Jan 28;10(2):e01247-24. doi: 10.1128/msystems.01247-24 (PMC11834404; doi:10.1128/msystems.01247-24)
Supplement: Supplemental material — Supplemental table and figures. [file msystems.01247-24-s0001.pdf]

List of supplemental table and figures

Table S1 Demographic characteristics of the collected 11 studies.

FIG S1 Rarefaction curve of collected sequencing data.

FIG S2 Scatter plot of delta age (predicted age - chronological age) in training set (A), test set (B) and OSCC prediction set (C).

FIG S3 Distribution of demographic characteristics of the 11 included studies. (A) Sample diagnostics. (B) Gender. (C) Sequencing region. (D) Sample type. (E) Geographical region.

FIG S4 Cross-study alterations in the oral microbiome during the progression of OSCC. A. Forest plots depicting alpha diversity differences between healthy and OPMD. B. Forest plots depicting alpha diversity differences between OPMD and OSCC. C. Differences between healthy and OPMD microbiome community. D. Differences between OPMD and OSCC microbiome communities.

FIG S5 29 differential OTUs (FDR ≤ 0.01) between healthy and OSCC samples across studies.

FIG S6 Similar microbiome changes in the saliva and rinse samples in response to OSCC.

Table S1 Demographic characteristics of the collected 11 studies

No.	1	2	3	4	5	6	7	8	9	10	11
Project No.	PRJNA870048	PRJNA813634	PRJNA756784	PRJNA412445	PRJEB39064	PRJNA751046	PRJNA700849	OEP000837	PRJEB37501	PRJNA421234	PRJNA386665
Year Pb	2023	2022	2022	2021	2021	2021	2021	2021	2021	2018	2017
Diagnose											
Healthy Control	90		39		25	9	8	12		7	127
OPMD					21				28		123
OSCC	91	25	20	17	27	9	2	30	43	8	124
Age (mean±SD)											
Healthy Control	59.3±6.5		37.4±12.5		46.8±9.1	44.2±9.1	51.5±7.9	NA		NA	52.3±14.2
OPMD					51.4±13.4				42.5±13.5		50.5±11.8
OSCC	63.1±11.1	54.4±10.6	49.4±10.2	57.3±11.1	53.3±11.2	56.8±14.9	69.5±5.5	61.3±12.1	57.7±11.0	63.9±10.4	53.3±10.9
Gender											
Male	129	17	42	8	73	10	9	17	71	6	338
Female	52	8	16	8		8	1	12		2	36
NA			1	1				13		7	
Tumor Site											
Gingiva	11	5	2			3		13	8		
Buccal mucosa	13	3	9			4		5	17	1	
Tongue	59	11	5			2	1	9	6	4	
Mouth floor	4	4						2		2	
Hard palate	4	2					1		1	1	
Buccal mucosa and gingiva			1								
NA			3	17	27			1	11		124
T Stage											
I				1		1		10		5	
II				6		4		18		2	
III				1		1	1	1			
IV				9		3	1			1	
NA	91	25	20		27			1	43		124
N Stage											
0				12		7	1	16		5	
1				4				8			
2				1							
2a										1	
2b							1	5		2	
3b						2					
NA	91	25	20		27			1	43		124

No.		1	2	3	4	5	6	7	8	9	10	11
Project No.		PRJNA870048	PRJNA813634	PRJNA756784	PRJNA412445	PRJEB39064	PRJNA751046	PRJNA700849	OEP000837	PRJEB37501	PRJNA421234	PRJNA386665
Clinical Stage												
	I				1	12	1		7	21	3	
	II				4	6	3		9	10	2	
	III				2	2	1	1	8	4		
	IV				10	7	4	1	5	8	3	
	NA	91	25	20					1			124
Pathological Grade												
	Well				9							
	Med				6							
	Poor				2							
	NA	91	25	20		27	9	2	30	43	8	124
Smoking												
	N	130				12	16		19		3	
	former					13			3		2	
	Y	51				48	2		6		2	
	NA		25	59	17			10	14	71	8	374
Drinking												
	N	143				30	18		21			
	former					5			1			
	Y	38				38			7			
	NA		25	59	17			10	13	71	15	374
Chewing Habits												
	N			19		11	13					
	former					30						
	Y			39		32	5			71		
	NA	181	25	1	17			10	42		15	374
Region		Eastern Asia	Eastern Asia	Southern Asia	Eastern Asia	Eastern Asia	Southern Asia	Latin America	Eastern Asia	Eastern Asia	Oceania	Eastern Asia

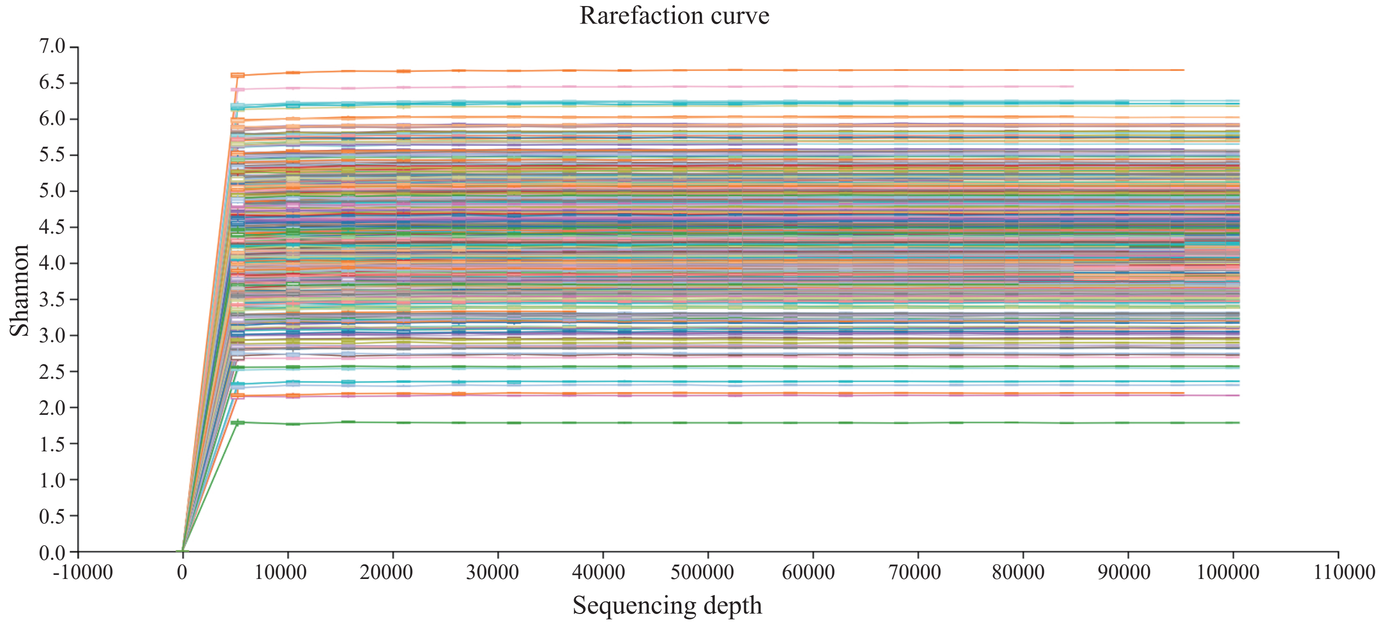

FIG S1 Rarefaction curve of collected sequencing data.

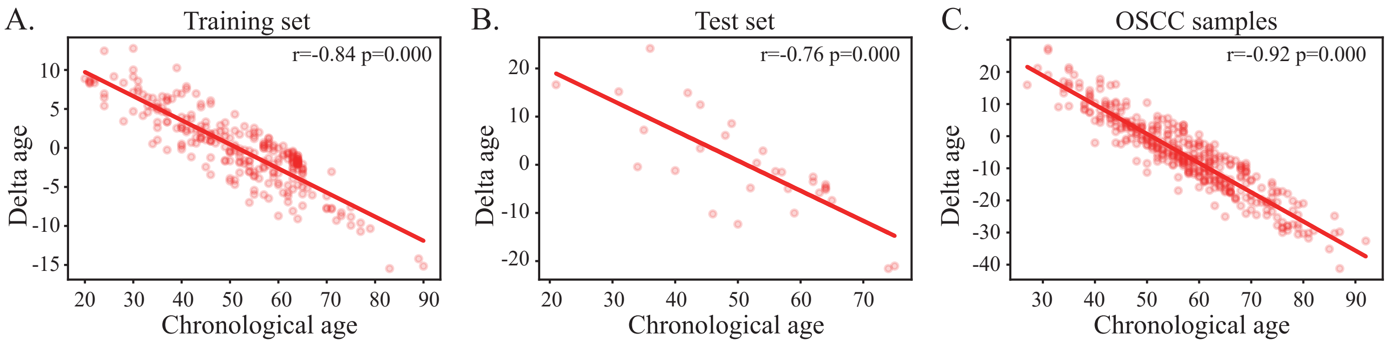

FIG S2 Scatter plot of delta age (predicted age - chronological age) in training set (A), test set (B) and OSCC prediction set (C).

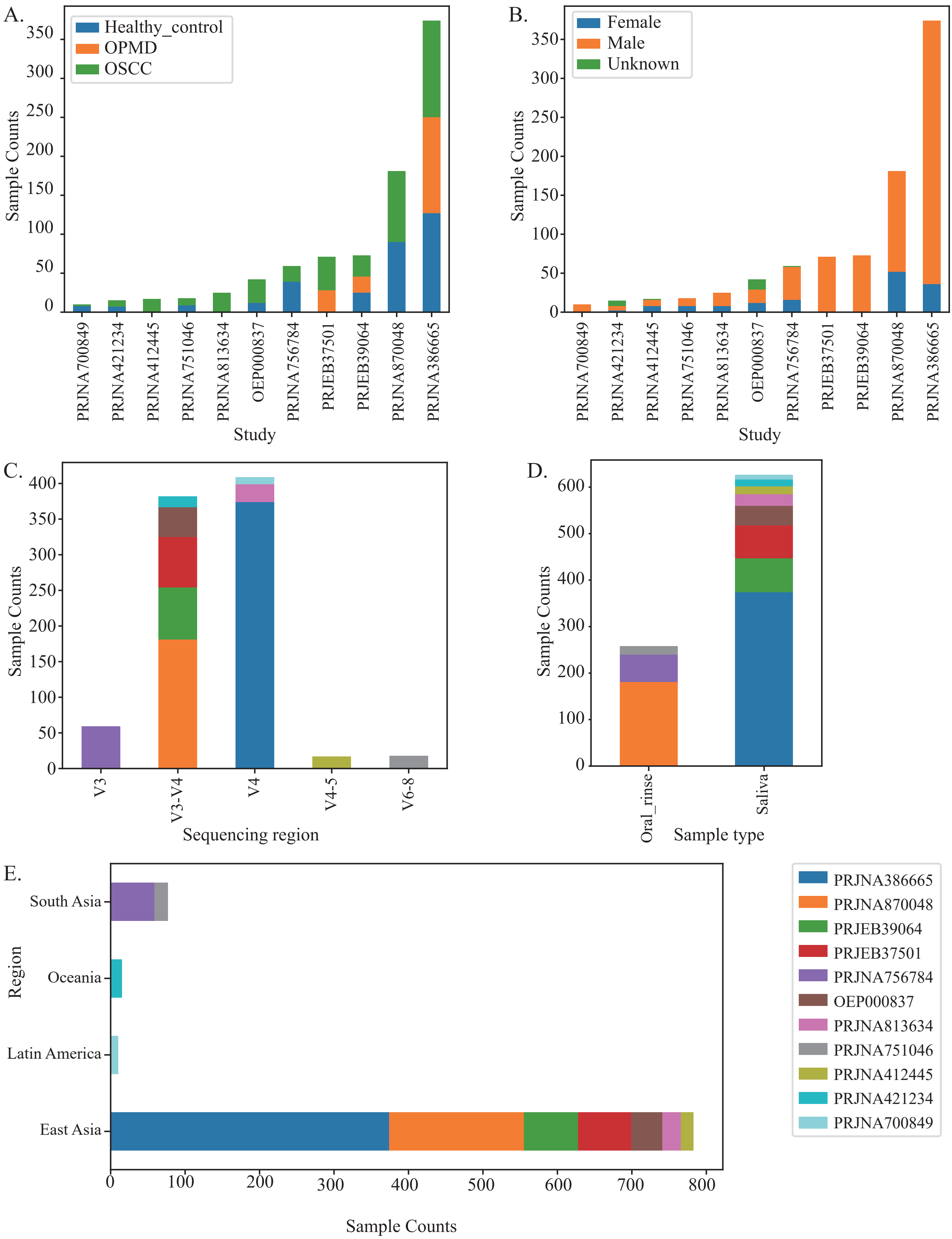

FIG S3 Distribution of demographic characteristics of the 11 included studies.
(A) Sample diagnostics. (B) Gender. (C) Sequencing region. (D) Sample type. (E) Geographical region.

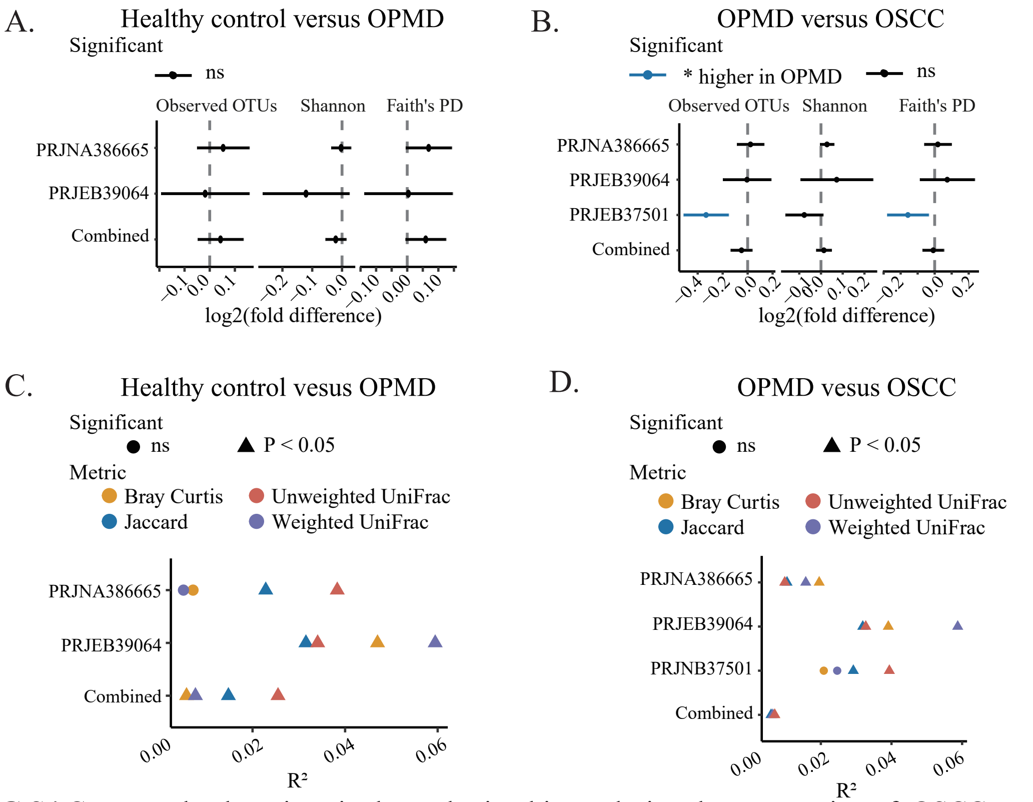

FIG S4 Cross-study alterations in the oral microbiome during the progression of OSCC. (A) Forest plots depicting alpha diversity differences between healthy and OPMD. (B) Forest plots depicting alpha diversity differences between OPMD and OSCC. (C) Differences between healthy and OPMD microbiome community. (D) Differences between OPMD and OSCC microbiome community .

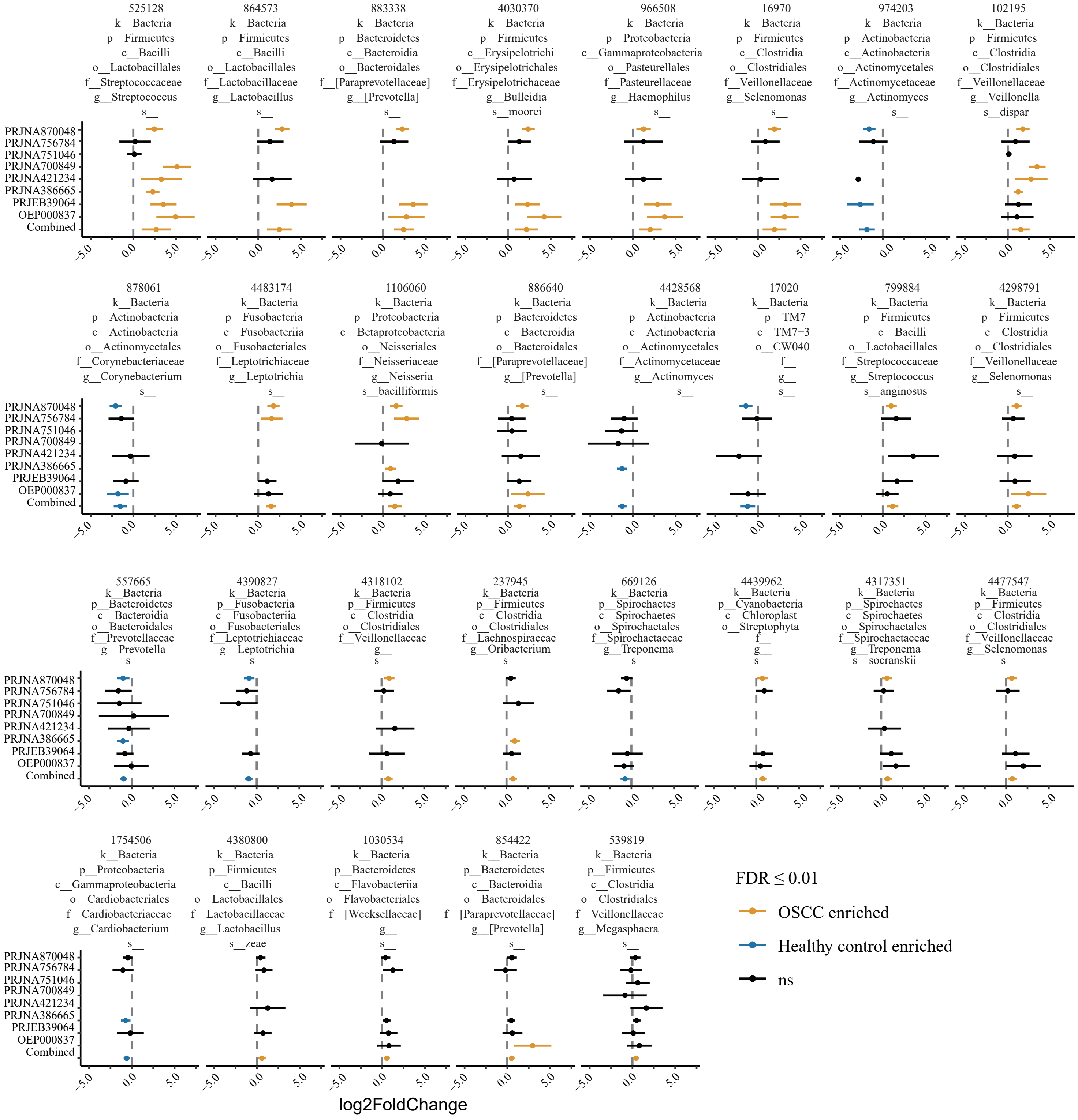

FIG S5 29 differential OTUs (FDR ≤ 0.01) between healthy and OSCC samples across studies.

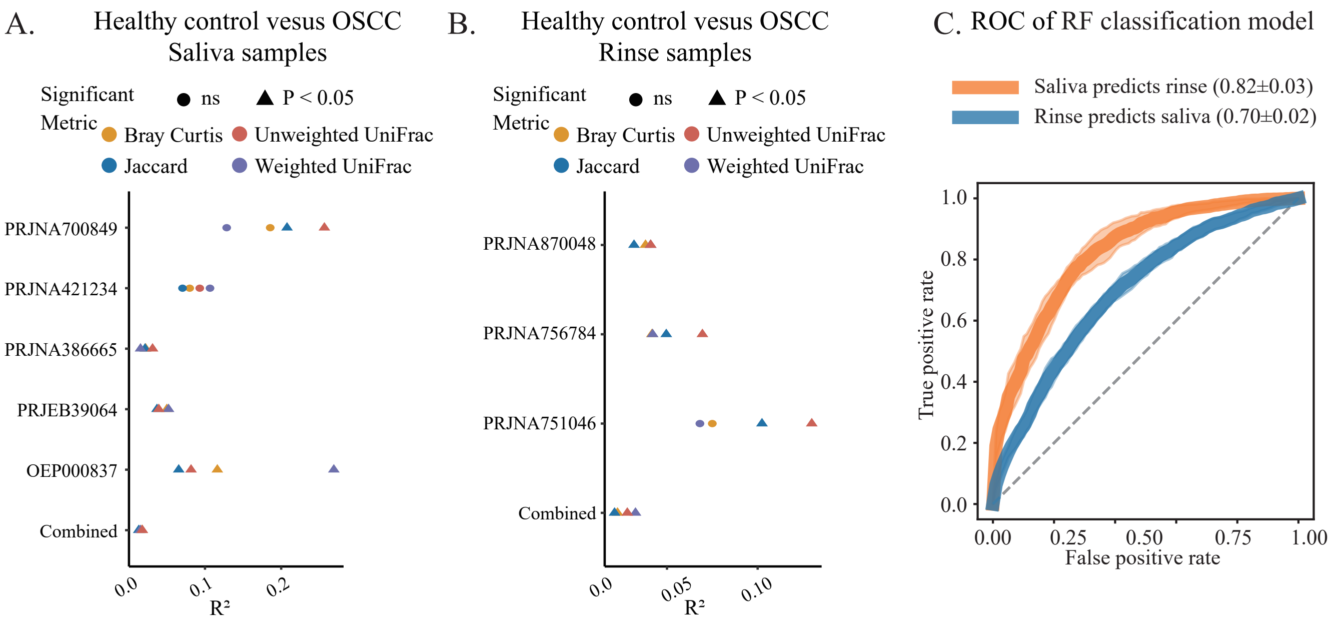

FIG S6 Similar microbiome changes in the saliva and rinse samples in response to OSCC.
(A-B) Differences between healthy and OSCC microbiome community in saliva(A) and rinse(B) samples were calculated using the ADONIS analysis based on Bray-Curtis, Jaccard, unweighted UniFrac, and weighted UniFrac distance metrics. The combined R^2 was computed by setting study as the stratifying factor in the ADONIS test. (C) The RF model was trained on the saliva microbiome to predict disease stage in rinse samples, and vice versa.
